# Use of regression models for development of a simple and effective biogas decision-support tool

**DOI:** 10.1038/s41598-023-32121-6

**Published:** 2023-03-27

**Authors:** Cuong Manh Duong, Teng-Teeh Lim

**Affiliations:** 1 Plant Science & Technology, University of Missouri, 147 Agricultural Engineering Building, Columbia, MO 65211-5200 USA; 2 Faculty of Biotechnology and Food Technology, Thai Nguyen University of Agriculture and Forestry, Thai Nguyen, Vietnam

**Keywords:** Biogas, Statistics, Mathematics and computing

## Abstract

Anaerobic digestion (AD) is an alternative way to treat manure while producing biogas as a renewable fuel. To increase the efficiency of AD performance, accurate prediction of biogas yield in different working conditions is necessary. In this study, regression models were developed to estimate biogas production from co-digesting swine manure (SM) and waste kitchen oil (WKO) at mesophilic temperatures. A dataset was collected from the semi-continuous AD studies across nine treatments of SM and WKO, evaluated at 30, 35 and 40 °C. Application of polynomial regression models and variable interactions with the selected data resulted in an adjusted R^2^ value of 0.9656, much higher than the simple linear regression model (R^2^ = 0.7167). The significance of the model was observed with the mean absolute percentage error score of 4.16%. Biogas estimation using the final model resulted in a difference between predicted and actual values from 0.2 to 6.7%, except for one treatment which was 9.8% different than observed. A spreadsheet was created to estimate biogas production and other operational factors using substrate loading rates and temperature settings. This user-friendly program could be used as a decision-support tool to provide recommendations for some working conditions and estimation of the biogas yield under different scenarios.

## Introduction

The total number of manure-fed anaerobic digestion (AD) plants in the United States has nearly doubled from 141 in 2010 to 273 in 2021^1^. The application of AD can be considered as an alternative way for manure treatment while biogas is produced as a renewable fuel. Biogas production generated from on-farm AD plants can be used for different purposes, such as heating or generating electricity. US-EPA^2^ estimated the energy potential of AD reactors fueled by swine manure (SM) could be 6,597,520 MWh per year, a significant factor for reducing farm operating costs. Many AD plants have been developed since 2009 because state-run programs, such as the Low Carbon Fuel Standard (LCSF) in California, pay credits directly to operators^3,4^. Economic benefits are responsible for the construction of many on-farm digesters^1^. However, if the estimation of biogas yield could not be scientifically estimated in advance, production might be higher than the biogas treatment capacity, possibly resulting in unintended emission. For instance, the emission of methane (CH_4_) from digesters or storage tanks into the atmosphere may occur when the biogas exceeds the storage or treatment capacity^5^. Accurate prediction of biogas production is needed before constructing new AD plants or even during the operation of the existing ones to avoid this environmental threat.

The application of mathematical models resulted in several trustworthy tools for estimating biogas production, such as the IWA Anaerobic Digestion Model No 1 (ADM1) developed in 1997^6^. ADM1 focused on biochemical and physico-chemical steps during biogas production, and methane yield could be estimated based on several initial parameters, including feedstock flowrate, total COD, alkalinity or pH^7^. Moreover, the kinetic and stoichiometric models, including the ADM1 mentioned above, are widely considered a reliable tool. They could be applied to estimate the reactor performance and the process stability under various conditions which could be far different from those adopted during the experiments for the model validation^8,9^. Machine learning or statistical learning has been used recently to develop biogas models, accounting for several factors and their relationships. A study conducted by Wang et al.^10^ focused on biogas prediction using machine learning algorithms. Eight parameters were selected for establishing the program, including glucan content, temperature, C/N ratio, total nitrogen, total carbon, lignin content, xylan content, and cellulose content. Biogas thermodynamics were predicted by applying multi-layer perceptron neural network and artificial neural network (ANN) in the work proposed by Farzaneh-Gord et al*.*^11^. Neural network models with ant colony optimization algorithms were used to predict biogas flow rate, as presented by Beltramo et al*.*^12^. Applications of neural network or ant colony algorithm were also reported in studies conducted by Dach et al.^13^, Nair et al*.*^14^ and Verdaguer et al*.*^15^. The use of machine learning to establish models is a new and promising way to predict biogas production with high accuracy. However, the application of these models was limited due to their complexity, which requires statistical skills and training.

The use of multiple regression is an attractive alternative for biogas prediction due to its simplicity and effectiveness^16^. Several regression models were established based on common factors including the type of substrates, feedstock loading rate, initial pH, etc. Lhanafi et al*.*^17^ studied co-digestion of dairy wastes using batch digesters at mesophilic conditions with temperatures at 38 ± 1 °C to investigate the relationship between three factors (pH, loading rate and inoculum) and biogas yield using an experimental design. Another model developed by Mao et al*.*^16^ was used to predict biogas production with two variables, initial pH and swine manure/corn straw (SM/CS) ratio. Data were collected from batch digesters using 1-L glass bottles under mesophilic conditions with three SM/CS ratios (30:70, 50:50, 70:30) and different initial pH values, ranging from 6.0 to 8.0. In another batch study conducted by Wang et al*.*^18^, dairy manure (DM) and chicken waste (CW) were co-digested under the mesophilic condition, in which five levels of DM/CW (14.6, 25, 50, 75, 85.4) and C/N (17.9. 20, 25, 30, 32.1) were selected for establishing nine treatments. Regression studies result in model equations that can be used to compare the accuracy of predicted versus actual methane yields. Although limited by the selection of input variable types and ranges, regression models are much easier than those created by machine learning. However, the effect of temperature was not focused in the models mentioned above, and they were based on results from batch studies, which represented the biogas potential of the substrates, rather than actual gas production in the long-term period. Batch experiments cannot simulate some common problems in the AD systems, such as overloading or improper substrate ratio, which, in some cases, may result in system disturbance or failure^19^. Continuous systems, in contrast, focus on the load of substrates for measuring biogas production consecutively, and can be used to evaluate actual biogas productivity and system performance in the long-run. Biogas models using data observed from continuous studies, considering temperature and other factors, are necessary for the estimation of AD production.

Results from previous studies showed the efficiency of combining SM and waste kitchen oil (WKO) as substrates for co-digestion in the AD systems^19^. The data also confirmed that organic loading rate (OLR), substrate ratio, and temperature were the main factors affecting biogas production, while the ratio of oil to manure was essential to maintain the balance of AD activity. In addition, a pilot study (unpublished data) showed that co-digestion of SM and WKO at the mesophilic condition (40 °C) was more efficient in terms of higher biogas production and more diverse microbial community compared to the thermophilic conditions, which was also reported by other studies^20,21^. Moreover, the operation of AD plants at mesophilic conditions is easier and requires less energy than those operated at higher temperatures^22^. Although co-digestion of SM and oily substrates have been studied previously, those publications were focused on a specific temperature instead of a range of values^23–25^. Studies at different mesophilic temperatures are crucial for the accurate evaluation of AD performance, in addition to developing prediction models for co-digesting SM and WKO. Moreover, a user-friendly tool is needed to predict biogas yields easily, an important factor in making decision for AD plant design and operation.

This study was conducted to investigate the relationship between biogas production and volatile solid (VS) loading rate of SM, oil-to-manure ratio, OLR, temperature, and pH at mesophilic conditions. Regression models were established to estimate the biogas production from three main variables—manure VS loading rate, oil-to-manure ratio and operating temperature specially for co-digestion of SM and WKO. Different approaches were applied to improve model accuracy. Finally, a spreadsheet was developed as a user-friendly decision-making tool for estimating biogas production using different inputs of manure, oil and temperature, as well as providing recommendations for AD plant construction and operation.

## Materials and methods

### Substrate collection and co-digestion set-up

The manure sample was picked up twice, in December 2019 and February 2021, from a central Missouri pig farm and stored at − 20 °C before use. Each batch of SM was tested for the TS and VS by EPA Method 1684^26^, with the VS values in the range of 25.0–29.1%. WKO (99.5% VS) was collected from campus dining services and kept at room temperature (24–25 °C). The fact that SM and WKO amount added was based on their VS and the stability of digester performance was observed when using a new batch of SM confirmed the relatively minimal effects of substrate characteristic changes on biogas production. Glass jars with a capacity of 1.9 L (0.5 gal) and a working volume of 1.4 L were selected as digesters. In addition, the 3.8-L jars were used when severe foaming and clogging issues were observed because of the high OLRs during the study (e.g., the digestion of 4 g-VS_SM_/L/day (M4) at 40 °C)^19^. The digesters were swirled manually three times per day and stored in a CO_2_ incubator model 3028 (Forma Scientific, Marietta, OH, USA) for temperature control. The hydraulic retention time (HRT) of 21 days and the two-day procedure for substrate loading and biogas measurement under normal room temperature was followed, based on similar studies conducted in the same laboratory^19,27^. When the 2-day bag’s volume was less than 4 L due to the low biogas production of the low-OLR treatments (e.g., M2), the gas measurement was only performed every-four days. Similarly, the bag measurement of the high-OLR digesters (e.g., M4 with 2 g-VS_WKO_/L/day) was conducted every day because of their high gas production. The methane yield was not analyzed because the study aimed to focus on total biogas production. The pH value was recorded using a Pinpoint meter (American Marine Inc, Ridgefield, CT, USA) as a simple indicator for measuring AD performance.

### Experimental design and data collection

Results from the previous study suggest that biogas production and AD stability depend on VS-loading of SM, temperature and oil-to-manure (O/M) VS ratio^19^, which was the primary reason for selecting these three variables in the regression model. Previous observations^19^ showed that: (1) loading more than 4 g-VS of SM per liter per day did not make a significant improvement to biogas yield; (2) the O/M ratio should not exceed 0.5; and (3) significant disturbance of biogas production was observed at the thermophilic condition (unpublished data). Therefore, VS loadings of SM in this study were selected as 2, 3 or 4 g-VS/L/day (M2, M3 or M4), temperatures in the range between 30 and 40 °C were focused and three levels of O/M VS ratios (0, 0.25 and 0.5 or R0, R0.25 and R0.5) were considered for model development. The three O/M ratios were chosen to represent the mono-digestion in which only SM was used (R0), the intermediate and the maximum levels of oil addition (R0.25 and R0.5). HRT was not evaluated in the model because a previous study conducted by Nogueira et al*.*^27^ showed an optimal HRT of 21 days, which was in agreement with the common range applied in complete mixed digesters^28^. In total, nine essays were set up in replicate which included combinations between the three VS loading levels of SM and three O/M ratios (Table 1). VS loading of oil was calculated based on the specific VS content of SM and O/M ratio in each essay. For example, M4R0.25 represented VS loadings of SM and WKO were 4 g-VS/L/day, and 4 × 0.25 or 1 g-VS/L/day, respectively.

**Table 1:** Experimental design in the study.

Essay	Factors				
	OLR_SM_	O/M VS ratio	OLR_WKO_	Total OLR	Temperature
	(g-VS/L/day)	–	(g-VS/L/d)	(g-VS/L/day)	(°C)
1	2	0	0	2.00	40, 35, 30
2	2	0.25	0.50	2.50	40, 35, 30
3	2	0.50	1.00	3.00	40, 35, 30
4	3	0	0	3.00	40, 35, 30
5	3	0.25	0.75	3.75	40, 35, 30
6	3	0.50	1.50	4.50	40, 35, 30
7	4	0	0	4.00	40, 35, 30
8	4	0.25	1.00	5.00	40, 35, 30
9	4	0.50	2.00	6.00	40, 35, 30

*OLR* organic loading rate, *SM* swine manure, *VS* volatile solid, *O/M* oil/manure.

The study started at 40 °C, then the temperature was decreased gradually to 35 °C and 30 °C. All essays were monitored for at least four HRTs at each temperature level to ensure the stability or failure of each treatment could be evaluated. Data were collected from the last two HRTs by averaging the biogas production of digesters in each group every-4 days to reduce error. Therefore, the dataset included 11 observations per essay per temperature level, except when the failure of digesters occurred in which biogas production was assigned as 0 mL/day. Data of M2R0, M2R0.5, M4R0.25 and M4R0.5 were adapted from a previous study conducted in triplicate^19^. Two other variables, OLR and pH were included in the dataset to measure their correlation with biogas production.

### Establishment and improvement of regression models

The dataset contained 247 observations, including one response variable (biogas production) and three key feature variables or predictors (VS loading of SM—X_1_, O/M ratio—X_2_, and Temperature—X_3_). The number of observations was less than expected (9 essays × 3 temperature levels × 11 observations/essay/temperature) because AD failures were observed in five combinations of loading rates and temperatures (M4R0.5 at 35 °C; M2R0.5, M3R0.5, M4R0.25 and M4R0.5 at 30 °C), reducing the data points collected in each treatment above from 11 to one. The correlation coefficients were calculated on every variable by using the package “ggally”^29^ in R software v4.2.0^30^. The results were used to evaluate the linear relationship between each pair of factors^31^. Next, a linear regression model was developed to estimate biogas production from three key variables, using the built-in function in R^32^. Because the “zero points” might have a negative impact on model performance^33^, another dataset was created by removing observations with biogas yield equal to 0. Variable correlation and linear regression models were performed again to compare the results in both scenarios (with the original or selected dataset) before further analysis.

Since the polynomial regression tends to fit the data better than a simple linear model^34^, second and third-order models with variable interactions were compared against linear regression. The significance of the models was determined by the *p-values* observed from the results. R-squared or adjusted R-squared was used to evaluate model performance because it is a more powerful statistic indicator than the others, such as mean squared error (MSE) or root mean square error (RMSE)^35^. Stepwise selection using package “olsrr” in R was applied to optimize the number of variables in the model which was performed by adding and removing variables after observing the changes^36–38^. Akaike information criterion was used to compare the performance of a model when processing stepwise procedure^39^. Additionally, the mean absolute percentage error (MAPE) was applied to determine model operation, as suggested by the literature^40,41^. Variance inflation factor (VIF) was evaluated by using package “car” as a criterion to analyze multicollinearity in the regression model^42^. Variable importance in the model was determined by the application of package “caret” in R^43^.

### Development of a user-friendly and on-farm AD tool for model application

Transferring experiment results from the lab to on-farm AD plants, biogas yield was assumed to be proportional to the digester’s working volume when the loading rate remained the same. For example, if the VS loading rate of SM was 2 g-VS/L/day (or 2 kg-VS/m^3^/day) and the biogas production of a digester with a working volume of 1.4 L was 2 L/day (or 1.45 L_biogas_/L_working_/day), then the biogas yield of an AD system with a working volume of 1000 m^3^ was supposed to be 1450 m^3^/d (or 1.45 m^3^/m^3^/day).

The model created was used to develop an Excel-based program to predict biogas production and provide recommendations for on-farm AD plants. The AD tool included three main components: Key variable input, AD variables and Model output. The Key variable requires the input of some parameters, including the number of pigs, manure production, and VS of manure and oil. In case the manure production is unknown, VS_SM_ production (kg-VS/day) would be estimated based on data reported by ASABE standard^44^, at 0.375 kg-VS/pig/day. Therefore, the total solid waste of a farm with 10,000 head of finishing pigs was supposed to be at 3750 kg-VS/day. If manure production was provided, VS production would be calculated, and all the recommendations would be based on this value. When specific values of digester size and manure loading rates were assigned, the maximum WKO loading rate would be determined to avoid system disturbance, based on the results of AD failures as reported above.

After specific values of each variable were entered based on the ranges recommended, the model output would provide information about biogas production, water loading rate or construction cost. The capital cost was assumed at $11.24/ft^3^ or $396.64/m^3^ for a complete mix digester, based on the study reported by Gloy^45^. In addition, the use of the on-farm AD tool needs to be based on the following model assumptions, including (1) Complete mix AD, (2) Pig manure as feedstock, (3) Co-digestion of swine manure and waste kitchen oil, (4) Hydraulic retention time of 21 days, and (5) No issue in ammonia content.

### Data analysis

Calculations of mean and standard deviation were performed using R software v4.2.0^30^. Development of linear and polynomial models was conducted with the use of built-in functions and packages in R^32^. The ANOVA (analysis of variance) function was applied to compare regression models^32^. Statistical significance was concluded when the *p-value* was less than 0.05. Community-contributed codes were applied and modified to create figures representing biogas productions^46,47^, variable correlations^29^ and variable importance^48,49^. Other figures and data analysis were performed using Excel (Microsoft Corporation, Redmond, WA, USA).

## Results and discussion

### Biogas production and relationship between variables

**Figure 1:**
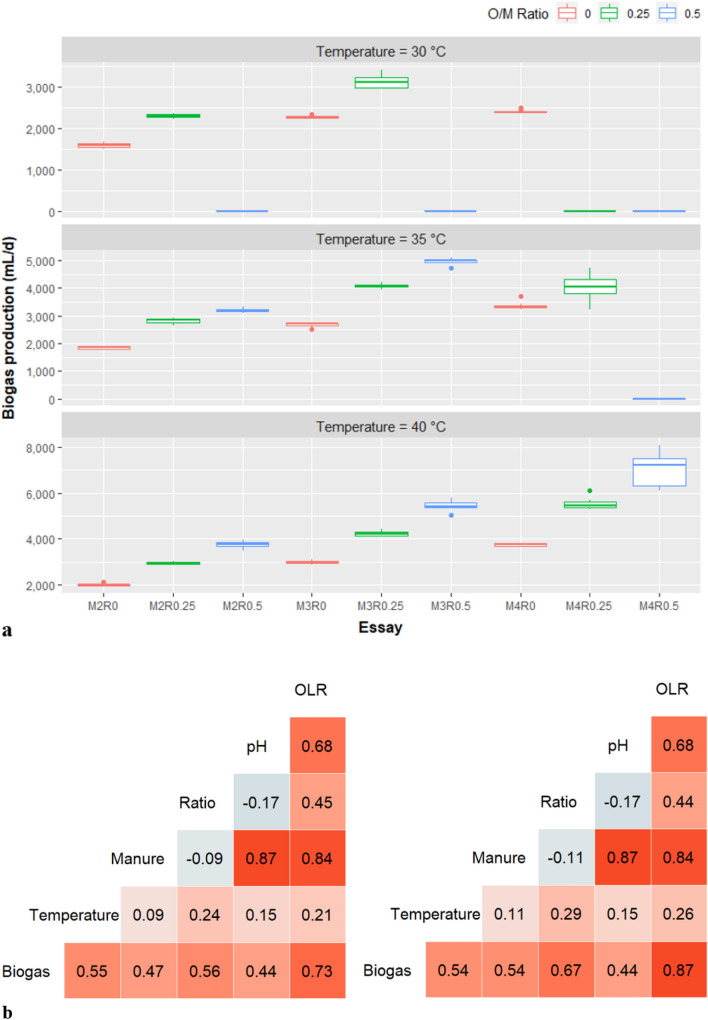
(**a**) Biogas production of each essay in different mesophilic temperatures and (**b**) correlation coefficients between biogas and other variables in original (left) and selected dataset (right). M2: organic loading rate (OLR) of swine manure = 2 g-VS/L/day; R0.25: VS ratio of swine manure/waste kitchen oil = 0.25; Ratio: O/M ratio.

Temperature showed a high impact on biogas production during the co-digestion of SM and WKO (Fig. 1a), suggesting that it could be an important factor to predict the reactor yield. In a typical mesophilic condition (40 °C), a stable expression of biogas production was observed in each treatment. High biogas yields were recorded in all essays at 40 °C, compared to lower temperature levels. However, the co-digestion of two feedstocks with high OLRs resulted in system failure when the temperature was decreased. At 35 °C, AD deterioration was observed in the M4R0.5 essay while more failures were reported at 30 °C. Besides M4R0.5, there were three more essays in which failures were recorded at the lowest temperature setting, including M2R0.5, M3R0.5, and M4R0.25. The O/M level of 0.5 was less effective at lower temperatures compared to other ratios. This suggests the importance of interactions between temperature and other factors while predicting biogas yield.

Correlation coefficients between biogas production and the key variables (SM loading rate, O/M ratio and temperature) ranged from 0.47 to 0.56 when the original data was applied (Fig. 1b). Moderate positive relationships among biogas yield and temperature and total OLR were observed (r = 0.55 and 0.73, respectively), suggesting the important roles of temperature and interactions between SM and substrate ratio in the model. Moreover, the removal of the failure points (when biogas production was zero) in the selected dataset increased the correlation coefficients of biogas and three main factors. The range of r was between 0.54 and 0.67 in the second scenario, implying that using the selected dataset might be appropriate to increase model accuracy. The high correlation between biogas production and OLR (r = 0.87) again suggests the interaction between SM and WKO might be necessary for model improvement. A low correlation between pH and biogas indicates that pH might not be a strong predictor to estimate biogas production. It is interesting to note that the high correlation between pH and VS loading of SM was recorded (r = 0.87 in both cases), which is in agreement with a study by Duan et al*.*^50^.

In the regression model with multiple variables, multicollinearity could occur if two or more variables were highly correlated, which could negatively impact the model interpretability^51,52^. In our study, manure loading and oil-to-manure ratio seems to be correlated. The low r score (− 0.09 and − 0.11 in two scenarios), however, showed a low correlation between these two factors. This was due to the selection of O/M ratios did not depend on SM levels, and indeed, it affected the OLRs of WKO rather than SM. Moreover, VIF values of three variables, a factor to evaluate multicollinearity^42^, resulted in scores from 1.02 to 1.08 in the simple regression model, lower than the threshold level of 5^52^. Again, it confirmed that multicollinearity was not a problem in this study. On the other hand, if SM, O/M ratio and OLR were included in the model, the VIFs of these factors were 39.37, 14.66 and 48.94, respectively, raising the concern of multicollinearity. This was in accordance with the fact that OLR was determined by both SM loading rate and oil-to-manure ratio, which caused the high correlations between these variables.

### Establishment of prediction models

A simple linear regression model was established based on the original dataset using 247 observations (Eq. 1) with a significant *p-value* (< 0.001). However, the adjusted R-squared was not high (0.7167), indicating that more than 71% of the biogas observations could be explained using this model^53^. The zero values of biogas production included in the dataset might be the reason for the moderate R-squared and adjusted R-squared^33^. All predictors were significant (*p* < 0.001) in the model. Based on estimations of the intercept and predictor’s coefficients listed in Table 2, the linear regression model was given as:

1 $${\text{Y }} = \, -\, {4822}.0{8 } + { 848}.{\text{56X}}_{{1}} + { 3647}.{9}0{\text{X}}_{{2}} + { 139}.0{\text{6X}}_{{3}} ,$$

where Y is biogas production (mL/day) and X_1_, X_2_, X_3_ are manure loading (g-VS/L/day), O/M ratio and temperature (°C), respectively.

**Table 2:** Estimation and significance of predictors in model for prediction of biogas production.

Variable	Estimation	Standard error	*p-value*
Intercept	− 4822.08	463.74	***
X_1_	848.56	61.00	***
X_2_	3647.90	254.59	***
X_3_	139.06	12.73	***

X_1_, X_2_, X_3_ represented VS loading rate of swine manure, waste kitchen oil/swine manure ratio and temperature, respectively.

Significance: ***p < 0.001, **p < 0.01, *p < 0.05.

#### Data pruning for improvement of linear regression model

Improvement of correlation coefficients between biogas production and O/M ratio or OLR when removing zero biogas points suggests that the process might increase the model accuracy. A new linear regression model created using the selected dataset without zero biogas samples resulted in a significant increase of adjusted R-squared, at 0.9015, compared to 0.7167 of the first one. This was in accordance with other studies when the dataset did not include zero values^33^. However, it should be kept in mind that AD failures were not evaluated in the new model and the estimation of biogas production at these conditions would be considered as an extrapolation, which may lead the prediction into a significant bias^54^.

#### Application of polynomial regression models and variable interaction

Application of quadratic (second-order) or cubic (third-order) term and interaction between key variables resulted in a significant model improvement, compared with the previous models. The new adjusted R-squared was 0.9656 in both cases, which was much higher than observed in the original model. Moreover, the MAPE scores of the two polynomial models were similar, at 4.16%, and were much lower than that of the previous model, at 9.86%. Some literature suggests that using polynomial regression increased the R-squared value and the model accuracy^34,55^. The *p-values* (< 0.001) showed statistical significance when applying both quadratic and cubic regression models. However, the R-squared and adjusted R-squared in the two cases were the same, and the ANOVA result showed no statistical difference between the two models. Therefore, the simpler or quadratic regression model with 10 predictors, including three key variables and their derivatives, was selected for the next round of model improvement.

**Figure 2:**
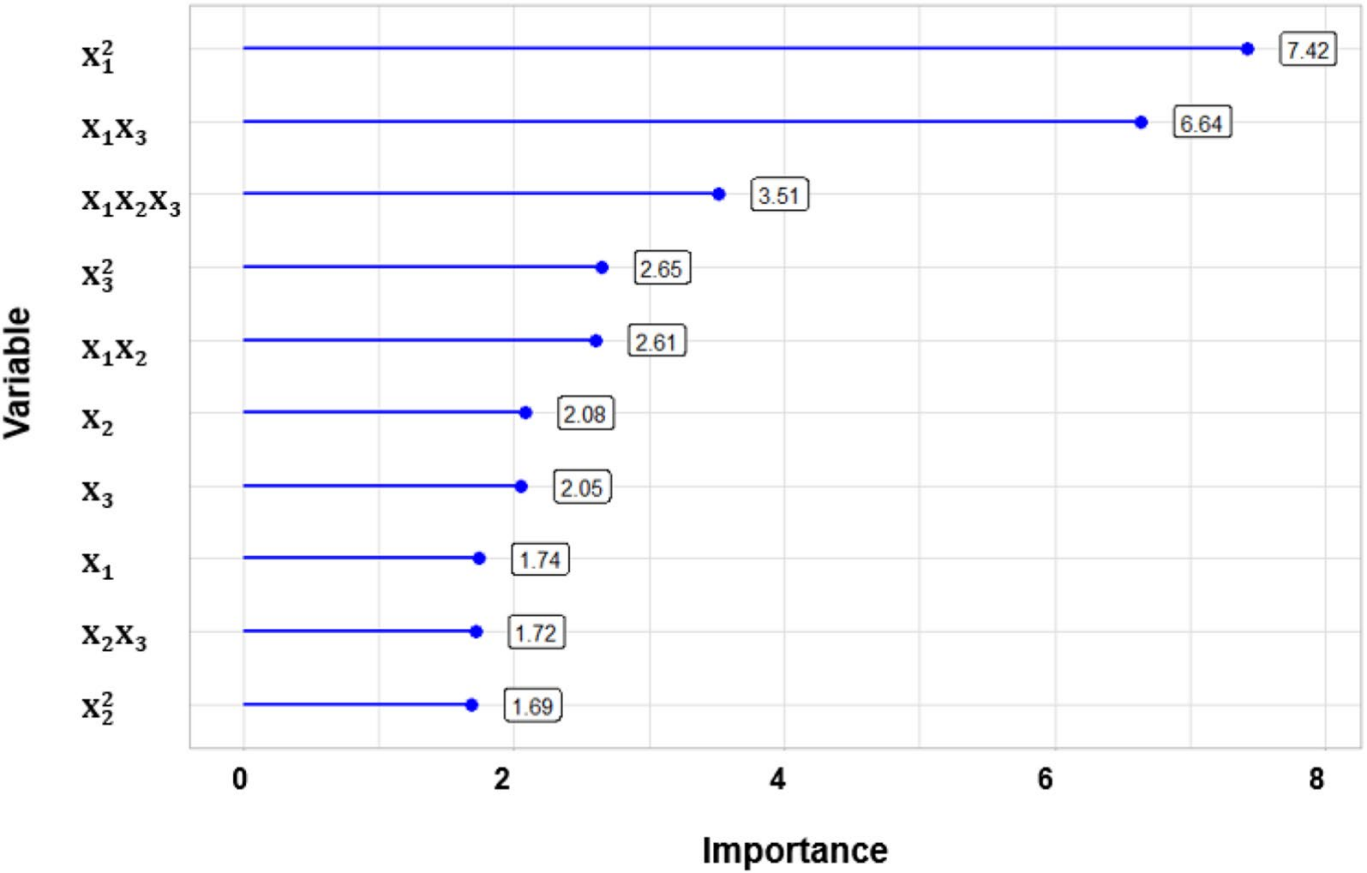
The importance score of each variable in the quadratic model. X_1_, X_2_, X_3_ represented VS loading rate of swine manure, waste kitchen oil/manure ratio and temperature, respectively.

Figure 2 demonstrated the importance of each variable in the model. The three most important variables were ${\mathrm{X}}_{1}^{2}$ and X_1_X_3_ and X_1_ X_2_X_3_, having scores from 7.42 to 3.51. This indicates the significance of manure loading and its interaction with temperature as well as the interaction of the three main factors to determine the biogas yield. Interestingly, the ratio squared and its interaction with temperature played the least significant role in the model with scores of 1.69 and 1.72, respectively.

When the stepwise algorithm was applied, X_1_ and X_2_ were removed from the model. The 8-variable model resulted in adjusted R^2^ at 0.9652, slightly lower than that of the 10-predictor model. No significant difference between the two models was observed (*p* = 0.0942). However, the removal of X_1_ and X_2_ led to the increase of MAPE to 4.24%, compared to 4.16% in the previous model. Therefore, the model with 10 variables was selected for further analysis. Among 10 predictors, seven showed statistical significance, except X_1_, ${\mathrm{X}}_{2}^{2}$ and X_2_X_3_ (Table 3). The model with all predictors were represented in Eq. (2), as:

2 $${\text{Y }} = \, - {4265}.{463 } + { 576}.{75}0{\text{X}}_{{1}} + { 7973}.{\text{188X}}_{{2}} + { 215}.{\text{762X}}_{{3}} {-}{ 265}.{\text{715X}}_{1}^{2} - {1}00{8}.{\text{368X}}_{2}^{2} - {3}.{\text{953X}}_{3}^{2} {-}{ 3565}.{\text{278X}}_{{1}} {\text{X}}_{{2}} + { 47}.{\text{611X}}_{{1}} {\text{X}}_{{3}} {-}{ 174}.{\text{484X}}_{{2}} {\text{X}}_{{3}} + { 126}.{\text{533X}}_{{1}} {\text{X}}_{{2}} {\text{X}}_{{3}} ,$$

where Y is biogas production (mL/day) and X_1_, X_2_, X_3_ are manure loading (g-VS/L/day), O/M ratio and temperature (°C), respectively.

**Table 3:** Estimation and significance of predictors in the quadratic model.

Variable	Estimation	Standard error	*p-value*
Intercept	− 4265.463	1957.119	*
X_1_	576.750	330.539	
X_2_	7973.188	3,827.445	*
X_3_	215.762	105.103	*
${\mathrm{X}}_{1}^{2}$	− 265.715	35.813	***
${\mathrm{X}}_{2}^{2}$	− 1008.368	597.580	
${\mathrm{X}}_{3}^{2}$	− 3.953	1.494	**
X_1_X_2_	− 3565.278	1367.367	**
X_1_X_3_	47.611	7.172	***
X_2_X_3_	− 174.484	101.598	
X_1_X_2_X_3_	126.533	35.998	***

*X*_1_, *X*_2_, *X*_3_ represented VS loading rate of swine manure, waste kitchen oil/manure ratio and temperature, respectively.

Significance: ***p < 0.001, **p < 0.01, *p < 0.05.

**Figure 3:**
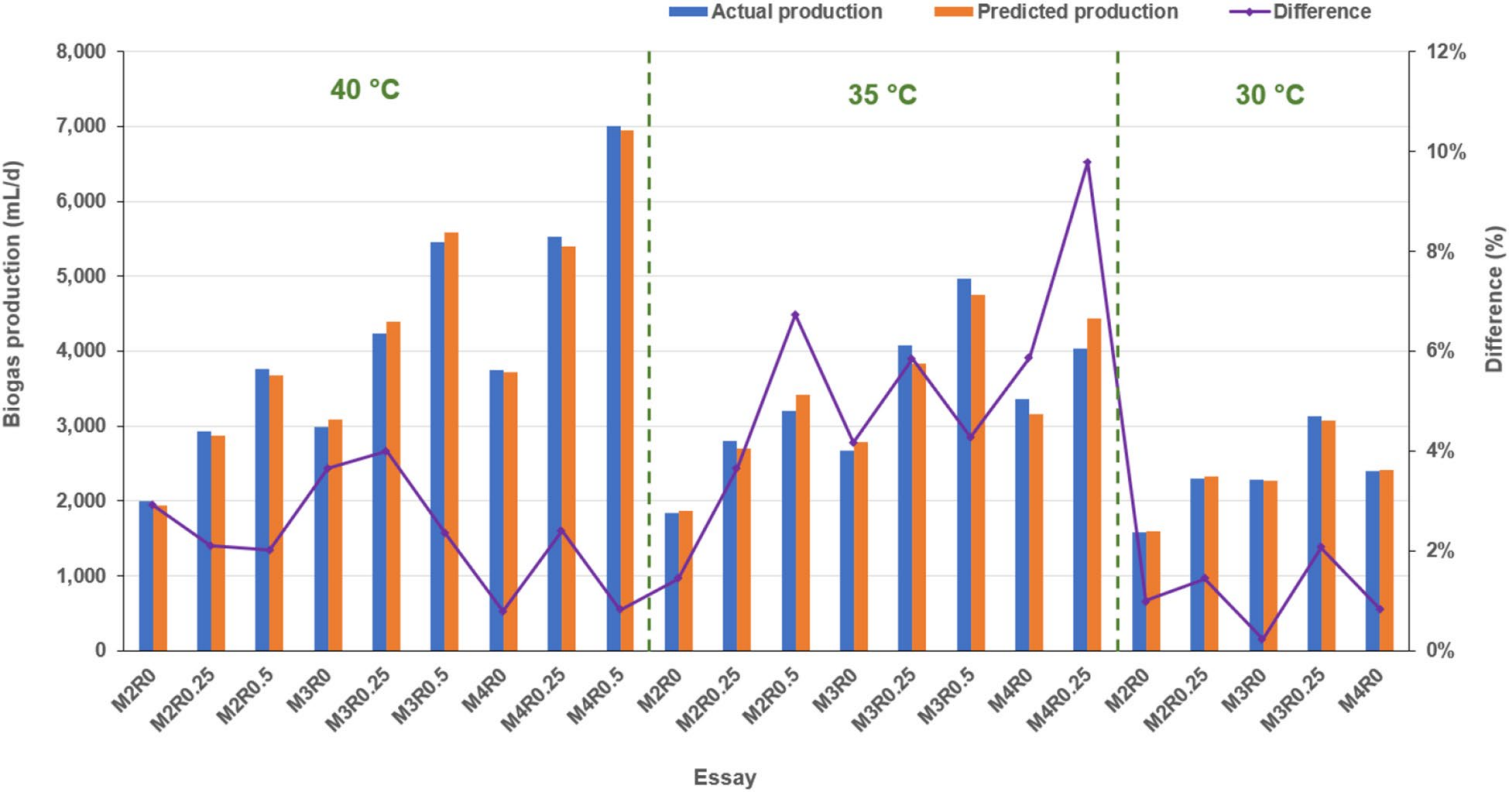
Comparison of predicted biogas production and average of actual data. M2: OLR of swine manure = 2 g-VS/L/day; R0.25: VS ratio of swine manure/waste kitchen oil = 0.25.

The comparison of biogas yields generated from the final model with the average of actual productions showed a high similarity, with the difference ranging from 0.2 to 6.7%, except the biogas yield of M4R0.25 at 30 °C, of which 9.8% difference was observed (Fig. 3). The results confirm the model’s accuracy in terms of biogas prediction and, therefore, the model could be useful for the estimation of the biogas production based on VS-loading of SM, O/M ratio and temperature. However, it should be noted that AD failure could not be predicted by the model due to the extrapolation after the removal of the zero biogas values.

### Decision support tool for model application

**Figure 4:**
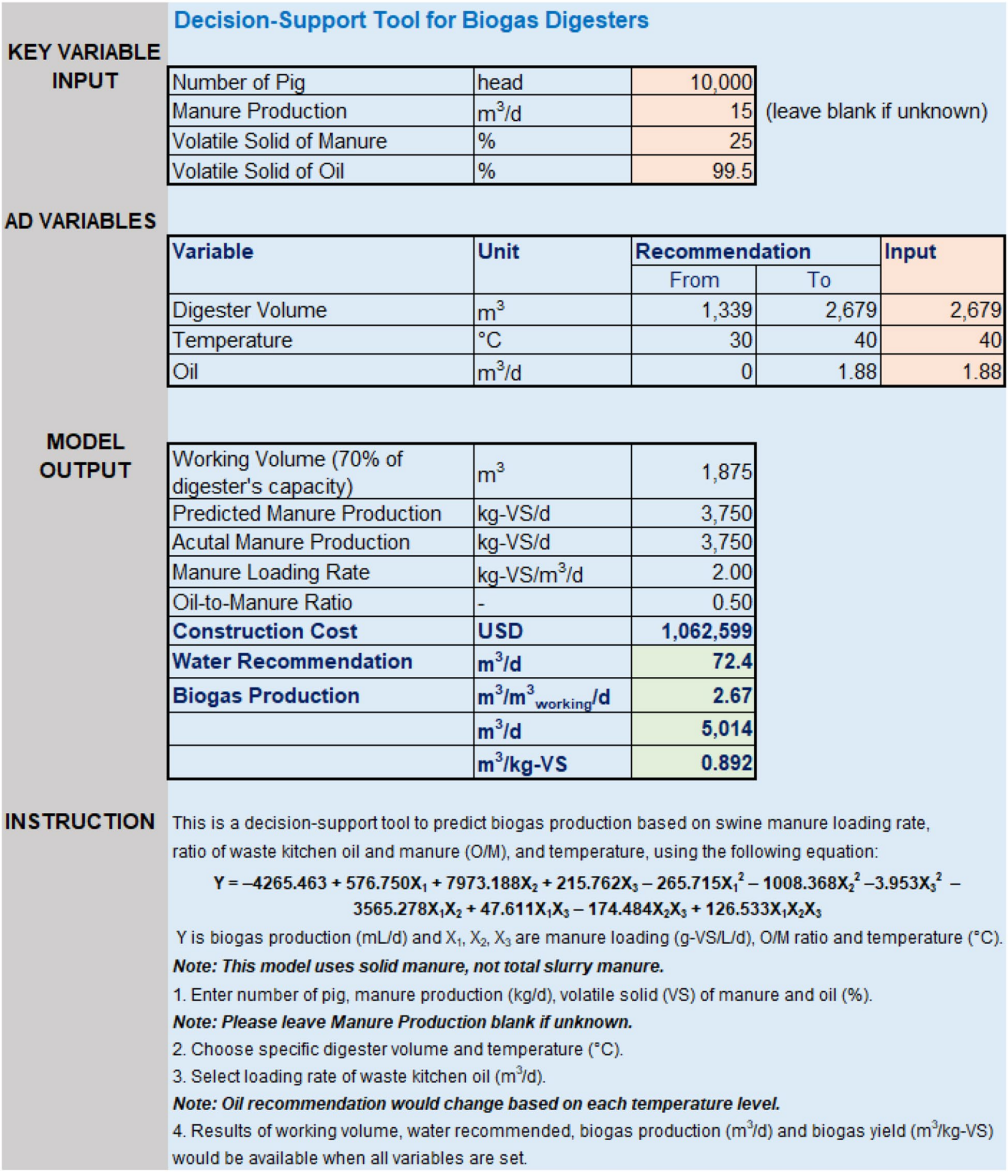
A user-friendly tool for recommendations of digester volume and other working conditions.

A user-friendly, Excel-based program was established as a decision-support tool for estimating biogas production based on SM and WKO loading and temperature, using the model developed for the on-farm application (Fig. 4). It could be used to provide recommendations for digester volume, oil loading and water usage before the construction of AD systems. For example, if SM production was 15 m^3^/day and VS_SM_ was 25.0%, daily VS production would be 3750 kg-VS/m^3^/day. Then, the digester size with a working volume of 70% its capacity would be recommended in the range of 1339 to 2679 m^3^, or from 47,286 to 94,608 ft^3^, which is similar to a typical size of a complete mix digester^56^. It is important to note that the relatively simple model and its recommendations depend on the results of the experimental setup and specific feedstocks used, which could be a limitation of the model performance.

Increasing digester capacity and working volume led to a decrease in VS_SM_ loading, resulting in expanding the ranges of other key variables (temperature and VS loading of WKO). More specifically, if the digester volume in the above example was 2679 m^3^ (maximum capacity), which was equal to the VS_SM_ loading of 2 kg-VS/m^3^/day, the temperature could be between 30 and 40 °C, and the addition of WKO loading could be up to 1.88 kg-VS/m^3^/day. On the other hand, a 1339-m^3^ co-digestion reactor, which was in accordance with the OLR_SM_ of 4 kg-VS/m^3^/day, should be operated at 35–40 °C, and WKO loading should be less than 0.94 m^3^/day if the temperature was set up at 35 °C. Biogas production (m^3^/day) would be calculated using the model after the values of each factor were determined. For example, if maximum values of digester volume (2679 m^3^), temperature (40 °C) and WKO (1.88 m^3^/day) were selected, which was equal to the OLR of M2R0.5, the biogas yield would be estimated at 5014 m^3^/day or 0.892 m^3^/kg-VS. Applying the models before operating on-farm experiments, therefore, could be an effective way to save time and effort.

#### Limitations of the prediction model and decision-support tool

Even though the accuracy was validated, the model could only work based on the specific ranges of each factor. For example, this study was conducted using three levels of temperatures, and there were no data for biogas production lower than 30 °C or higher than 40 °C when developing the models. Moreover, VS loading rate of SM less than 2 g-VS/L/d was not included in the study. In general, while OLRs of large-scale digesters could be higher than that level, some are operated with an even lower loading rate^57,58^. Using the model to predict biogas production based on the values out of the recommended ranges of each factor could be considered as an extrapolation and may result in a significant bias^54^. Further studies should focus on different working conditions so that the prediction ranges of the model could be expanded. It should be noted that the kinetic and stoichiometric models, as discussed above, should be considered as a more reliable tool for determining the system performance and stability when more operating variables are included. Those models are widely accepted by engineers for designing the biogas reactors, and the longer development and iterations of calibration would provide a better model accuracy while considering many important factors which were not addressed in this study, such as the change of HRT^7–9^.

Although the final model showed high R-squared and adjusted R-squared values, it was based on laboratory studies. No data was collected from on-farm AD plants for comparing predicted results with actual biogas production. Meanwhile, several factors associated with on-farm AD reactors could affect biogas production, such as design and type of digesters, HRT, fluctuation of temperature, change of animal diet, etc.^59–61^, that might reduce the model accuracy. Therefore, a comparison of data generated from the model with actual biogas productions is necessary to improve the model performance for the on-farm application. Other approaches, such as generalized linear models or Tweedie generalized linear model should be considered to create prediction models with better R-squared values, hence improving the model accuracy^62^.

## Conclusion

The developed model showed an effective way to predict biogas production based on SM loading rate, O/M ratio, and temperature in mesophilic AD systems. Application of the selected dataset and quadric regression with variable interactions significantly increased the adjusted R-squared value to 0.9656, compared with a lower value at 0.7167 from the linear regression model using the original dataset. The significance of the 10-variable, quadric model produced a MAPE score of 4.16%. In addition, the use of an Excel-based program made it convenient for farm owners to estimate biogas production when focusing on certain values of each factor. However, it should be kept in mind that the model could not predict the system failure, and the factors’ range was limited. Model calibration by comparing predicted data with biogas yields generated from actual AD plants or other models should be considered to improve the model accuracy.

## Supplementary Information

Supplementary Information 1.

Supplementary Information 2.

## Data Availability

The datasets and R codes used and analyzed during the current study are included in the Supplementary Information. Other materials are available from the corresponding author on reasonable request.
