# Supplementary material for: Use of regression models for development of a simple and effective biogas decision-support tool
Source: Sci Rep. 2023 Mar 27;13:4933. doi: 10.1038/s41598-023-32121-6 (PMC10042808; doi:10.1038/s41598-023-32121-6)
Supplement: Supplementary file 2 — Supplementary Information 2. [file 41598_2023_32121_MOESM2_ESM.docx]

**Use of regression models for development of a simple and effective biogas decision-support tool**

Cuong Manh Duong^a,b,*^, Teng-Teeh Lim^a^

^a^ Plant Science & Technology, University of Missouri, Columbia, MO 65211-5200, USA

^b^ Faculty of Biotechnology and Food Technology, Thai Nguyen University of Agriculture and Forestry, Thai Nguyen, Vietnam

^*^ Corresponding author: 147 Agricultural Engineering Building, University of Missouri, Columbia, MO 65211-5200, USA.

Email address: [duongmanhcuong@tuaf.edu.vn](mailto:duongmanhcuong@tuaf.edu.vn)

**The R codes for analyzing the dataset and developing models.**

**#Dataset:**

gas1=read.csv("~/Supplementary Dataset.csv")

**## Creation of boxplot**

library(ggplot2)

ggplot(gas1, aes(x=Essay, y=Biogas, color=factor(Ratio))) +

geom_boxplot() +

facet_wrap(~Temperature, scales="free_y",ncol=1,

labeller = labeller(Temperature=c(`40`="Temperature = 40 °C", `35`="Temperature = 35 °C", `30` = "Temperature = 30 °C")))+

labs(color="O/M Ratio", y="Biogas production (mL/d)")+

theme(legend.position = "top",

legend.justification = "right",

axis.title.x = element_text(color="Black", size=12, face="bold",vjust = -1),

axis.title.y = element_text(color="Black", size=12, face="bold",vjust = +3.5),

strip.text=element_text(size=11),

legend.margin = margin(0, 0, 0, 0))+ scale_y_continuous(labels=scales::comma)

**#Determination of correlation coefficients**

library(ggcorrplot)

library(GGally)

ggcorr(gas1,size = 8, label = TRUE,label_round = 2, hjust = 0.7, label_size = 8)

ggcorr(subset(gas1, Biogas>0),size = 8, label = TRUE,label_round = 2, hjust = 0.7, label_size = 8)

###############################

**#Development of model based on original dataset:**

gas1.md1 = lm(Biogas ~ Manure + Ratio + Temperature, data = gas1)

summary(gas1.md1)

**#Determination of Variance inflation factor (VIF)**

library(car)

**#The simple model with three variables:**

vif(gas1.md1)

**#The simple model with Manure, O/M ratio, Temperature and OLR:**

vif(lm(Biogas ~ Manure + Ratio + Temperature + OLR, data = gas1))

###############################

**# Development of models based on selected dataset:**

library(dplyr)

gas2 = filter(gas1, Biogas > 0)

gas2.md1 = lm(Biogas ~ Manure + Ratio + Temperature, data = gas2)

summary(gas2.md1)

**#The second and third-order models:**

gas2.md3 = lm(Biogas ~ Manure + Ratio + Temperature + I(Manure^2) + I(Ratio^2) + I(Temperature^2) + Manure*Ratio*Temperature, data = gas2)

summary(gas2.md3)

gas2.md4 = lm(Biogas ~ Manure + Ratio + Temperature + I(Manure^3) + I(Ratio^3) + I(Temperature^3) + Manure*Ratio*Temperature, data = gas2)

summary(gas2.md4)

**#Comparison of the second and third-order models:**

anova(gas2.md3,gas2.md4)

**#Determination of variable importance:**

library(caret)

Imp = as.data.frame(varImp(gas2.md3))

Imp = data.frame(Variable = c("X1","X2","X3","X1^2","X2^2","X3^2","X1X2","X1X3","X2X3","X1X2X3"),

Importance = Imp$Overall)

Imp

ggplot(Imp, aes(x=reorder(Variable,Importance), y=Importance)) +

geom_point( color="Blue", size=3)+

geom_segment( aes(x=Variable, xend=Variable, y=0, yend=Importance),

color='Blue', size=1) +

labs(x="Variable", y="Importance")+

coord_flip() +

theme_light() +

geom_label(aes(Variable, Importance , label = signif(round(Importance, digits = 2))),

nudge_y = 0.45, size = 4)

**#Stepwise procedure for variable selection:**

library(olsrr)

ols_step_both_aic(gas2.md3)

olsrr::ols_step_both_aic(gas2.md3, details = TRUE)

**#Determination of mean absolute percentage error (MAPE):**

library(ie2misc)

mape(predict(gas2.md1, gas2), gas2$Biogas, na.rm = FALSE)

mape(predict(gas2.md3, gas2), gas2$Biogas, na.rm = FALSE)
